# Cochlea-Specific Deletion of Ca_v_1.3 Calcium Channels Arrests Inner Hair Cell Differentiation and Unravels Pitfalls of Conditional Mouse Models

**DOI:** 10.3389/fncel.2019.00225

**Published:** 2019-05-22

**Authors:** Stephanie Eckrich, Dietmar Hecker, Katharina Sorg, Kerstin Blum, Kerstin Fischer, Stefan Münkner, Gentiana Wenzel, Bernhard Schick, Jutta Engel

**Affiliations:** ^1^Department of Biophysics, Center for Integrative Physiology and Molecular Medicine (CIPMM), School of Medicine, Saarland University, Homburg, Germany; ^2^Department of Otorhinolaryngology, Saarland University, Homburg, Germany

**Keywords:** inner hair cell, Ca^2+^ channel, Ca_v_1.3, BK, conditional knockout, *flex* switch, GFP toxicity, hearing

## Abstract

Inner hair cell (IHC) Ca_v_1.3 Ca^2+^ channels are multifunctional channels mediating Ca^2+^ influx for exocytosis at ribbon synapses, the generation of Ca^2+^ action potentials in pre-hearing IHCs and gene expression. IHCs of deaf systemic Ca_v_1.3-deficient (Ca_v_1.3^-/-^) mice stay immature because they fail to up-regulate voltage- and Ca^2+^-activated K^+^ (BK) channels but persistently express small conductance Ca^2+^-activated K^+^ (SK2) channels. In pre-hearing wildtype mice, cholinergic neurons from the superior olivary complex (SOC) exert efferent inhibition onto spontaneously active immature IHCs by activating their SK2 channels. Because Ca_v_1.3 plays an important role for survival, health and function of SOC neurons, SK2 channel persistence and lack of BK channels in systemic Ca_v_1.3^-/-^ IHCs may result from malfunctioning neurons of the SOC. Here we analyze cochlea-specific Ca_v_1.3 knockout mice with green fluorescent protein (GFP) switch reporter function, *Pax2::cre;Cacna1d-eGFP*^flex/flex^ *and Pax2::cre;Cacna1d-eGFP*^flex/-^. Profound hearing loss, lack of BK channels and persistence of SK2 channels in *Pax2::cre;Cacna1d-eGFP*^flex/-^ mice recapitulated the phenotype of systemic Ca_v_1.3^-/-^ mice, indicating that in wildtype mice, regulation of SK2 and BK channel expression is independent of Ca_v_1.3 expression in SOC neurons. In addition, we noticed dose-dependent GFP toxicity leading to death of basal coil IHCs of *Pax2::cre;Cacna1d-eGFP*^flex/flex^ mice, likely because of high GFP concentration and small repair capacity. This and the slower time course of *Pax2*-driven Cre recombinase in switching two rather than one *Cacna1d-eGFP^flex^* allele lead us to study *Pax2::cre;Cacna1d-eGFP*^flex/-^ mice. Notably, control *Cacna1d-eGFP^flex/-^* IHCs showed a significant reduction in Ca_v_1.3 channel cluster sizes and currents, suggesting that the intronic construct interfered with gene translation or splicing. These pitfalls are likely to be a frequent problem of many genetically modified mice with complex or multiple gene-targeting constructs or fluorescent proteins. Great caution and appropriate controls are therefore required.

## Introduction

The L-type calcium (Ca^2+^) channel Ca_v_1.3 is the main voltage-gated Ca^2+^ channel (VGCC) in inner hair cells (IHCs) and essential for hearing (Platzer et al., 2000; Baig et al., 2011). In both pre-hearing and mature IHCs, voltage-activated Ca_v_1.3 channels trigger glutamate release resulting in signal transmission to the auditory nerve (Brandt et al., 2003). Before the onset of hearing at postnatal day 12 in mice, IHCs produce spontaneous Ca^2+^-action potentials (Kros et al., 1998; Platzer et al., 2000; Marcotti et al., 2003) required for the terminal differentiation of IHCs (Brandt et al., 2003; Nemzou et al., 2006; Johnson et al., 2013a) and maturation of the auditory brainstem (Tritsch and Bergles, 2010; Clause et al., 2014; Babola et al., 2018). IHC spontaneous activity is modulated by transient efferent input originating in the superior olivary complex (SOC), which activates small-conductance SK2 potassium (K^+^) channels and thereby causes hyperpolarization of the IHC membrane potential (Glowatzki and Fuchs, 2000; Oliver et al., 2000). Around the onset of hearing, IHCs loose their efferent input (Simmons, 2002), SK2 channels are down-regulated (Marcotti et al., 2004) and spontaneous activity ends with the up-regulation of BK and KCNQ4 K^+^ channels (Kros et al., 1998; Oliver et al., 2003). IHCs of systemic Ca_v_1.3^-/-^ mice fail to acquire a mature composition of K^+^ channels (Brandt et al., 2003; Nemzou et al., 2006), which is likely caused by lack of spontaneous activity and impaired Ca^2+^-dependent transcriptional regulation. However, altered efferent modulation due to lack of Ca_v_1.3 in brainstem nuclei might add to the phenotype. Ca_v_1.3 plays an intrinsic role for development and function of SOC neurons (Hirtz et al., 2011, 2012; Satheesh et al., 2012) and is therefore regarded not only as a peripheral but also a central deafness gene (Willaredt et al., 2014).

Here, the effects of cochlea-specific ablation of Ca_v_1.3 channels before birth on the electrophysiological and molecular phenotype of IHCs as well as hearing function were investigated. To this end, *Cacna1d-eGFP^flex^* mice were used, in which the ablation of *Cacna1d* encoding Ca_v_1.3 channels is directly coupled to the expression of the reporter gene *eGFP* via Cre-induced inversion (“*switch*”) of the floxed allele (Satheesh et al., 2012). They were crossed with *Pax2::cre* mice (Ohyama and Groves, 2004), where Cre expression is initiated at E9.5 in the otic vesicle (Lawoko-Kerali et al., 2001; Burton et al., 2004) and found in the mature organ of Corti and spiral ganglion neurons (SGN) but not in the nuclei that are part of the afferent-efferent feedback loop onto hair cells, i.e., ventral cochlear nucleus and the SOC (Zuccotti et al., 2012).

## Materials and Methods

### Animals

*Cacna1d-eGFP^flex^* mice were generated within the *CavNET* consortium (EU-CAVNET MRTN-CT-2006-035367) by Katrin Bartels née Kunert, Kai Schönig and Dusan Bartsch, Central Institute of Mental Health, Mannheim, Germany (Satheesh et al., 2012). They were cross-bred with *Cacna1d*^-/-^ mice (Platzer et al., 2000) and *Pax2::cre* mice (Ohyama and Groves, 2004; Zuccotti et al., 2012). To reduce the risk of unwanted effects caused by Cre expression, only mice heterozygous for *Pax2::cre* were used (Jae Huh et al., 2010; Janbandhu et al., 2014). Animals were housed with free access to food and water at an average temperature of 22°C and a 12 h light-dark cycle. Mice of either sex were sacrificed by decapitation under isoflurane anesthesia and their cochleae were dissected from the temporal bones. All experiments were carried out in accordance with the European Communities Council Directive (86/609/EEC) and approved by the regional board for scientific animal experiments of the Saarland, Germany. Additional ethics approval was not required according to the local and national guidelines.

### Genotyping

*The Pax2::cre* allele was genotyped using primers detecting *Cre*: 5′-GCC TGC ATT ACC GGT CGA TGC AAC GA-3′ and 5′-GTG GCA GAT GGC GCG GCA ACA CCA TT-3′ (product size: 726 bp). The *Cacna1d-eGFP^flex^* (“*flex*”) allele was identified using: 5′-TTC AAG GAC GAC GGC AAC TAC AAG-3′ and 5′-CGG CGG CGG TCA CGA ACT CC-3′ (product size: 380 bp). To exclude accidental occurrence of unwanted embryonal or germline recombination of the *flex* allele in pups without Cre, we regularly used the following primers: “*Flex A*” (5′-GGA GTT GTG TAT ATC TGT TAA GCC ATG-3′), “*Flex B*” (5′-GCT GTT GGG CTG AGA AGT TGG T-3′) and “*Flex C*” (5′-CCA GAA GAT TCC ACT AAA GGT CAT-3′), detecting wildtype (*A-B* band, ∼450 bp), intact *flex* (*A-B* band, ∼600 bp) and switched *flex* allele (*B-C* band, ∼700 bp) (Bartels, 2009). The *Cacna1d*^-^ (“Ca_v_1.3^-^”) allele was genotyped with the primers: “*Ca_v_1.3 sense (s)*” (5′-GCA AAC TAT GCA AGA GGC ACC AGA-3′), “*Ca_v_1.3 antisense (as)*” (5′-TAC TTC CAT TCC ACT ATA CTA ATG CAG GCT-3′) and “*Ca_v_1.3 neosense (ns)*” (5′-TTC CAT TTG TCA CGT CCT GCA CCA-3′) yielding a wildtype (*s-as*, ∼300 bp) and/or a knockout band (*s-ns*, ∼450 bp).

### Hearing Measurements

Auditory brainstem responses (ABR) and distortion product otoacoustic emissions (DPOAE) were recorded in anesthetized mice aged 4–6 weeks as described in Fell et al. (2016). Growth functions of ABR waves I to IV in response to click stimuli were analyzed for peak-to-peak amplitudes and latencies between the click stimulus delivered at *t* = 0 and the time point of the negative peak of the respective wave.

### Electrophysiological Recordings

Apical-turn organs of Corti were acutely dissected from young adult mice (P17–P23) in solution containing (in mM): 70 lactobionate⋅NaOH, 83 NaCl, 10 HEPES, 5.8 KCl, 5.3 glucose, 1.3 CaCl_2_, 0.95 MgCl_2_, 0.7 NaH_2_PO_4_. For Ba^2+^ current recordings, the bath solution contained (in mM): 72.5 lactobionate⋅NaOH, 40 NaCl, 35 TEA, 15 4-AP, 10 BaCl_2_, 10 HEPES, 5.6 glucose, 0.9 MgCl_2_. Both solutions were adjusted to pH 7.35, 320 mosmol kg^-1^. Quartz pipettes were used and filled with (in mM): 112 Cs^+^ methane sulfonate, 20 CsCl, 10 Na^+^ phosphocreatine, 5 HEPES, 1 EGTA, 4 MgCl_2_, 4 Na_2_ATP, 0.3 GTP, 0.1 CaCl_2_. Pipette solution was adjusted to pH 7.36, 305 mosmol kg^-1^.

Before performing whole-cell patch clamp recordings using an Optopatch (Cairn Research, United Kingdom) or an Axopatch 200B amplifier (Molecular Devices, United States), green fluorescent protein (GFP) fluorescence of the specimen was assessed with an epifluorescence system consisting of a UV lamp and FITC fluorescence filters attached to the patch microscope (Olympus BX51WI with a 40 x water immersion objective, Germany) and a CCD camera (Scientifica, United Kingdom). Ba^2+^ currents were elicited by depolarizing the cells for 8 ms from –98 to +48 mV in 5 mV increments. Uncompensated series resistance was corrected by 70–80%. Analysis, including off-line linear leak subtraction and correction of the currents by subtracting the liquid junction potential of 8 mV, was performed using Igor Pro software (Wavemetrics, United States). *I–V* relations were calculated as the average current taken from the last ms of the voltage step as a function of the respective voltage.

*I–V* curves of Ba^2+^ currents were fitted to a second-order Boltzmann function times Goldman-Hodgkin-Katz driving force to determine parameters of activation, the voltage of half-maximum activation, *V_h_*, and the voltage sensitivity of activation determined by the slope factor *k*, according to

(1) $$I=-P_{\max}\text{ z F }\nu (\frac{{\left[Ba\right]}_{o}}{e^{\nu -1}}+\frac{{\left[Ba\right]}_{i}}{e^{-\left(\nu +1\right)}})\cdot {\left(\frac{1}{1+e^{\frac{\left(V_{h}-V\right)}{k}}}\right)}^{2}$$

where *I* is *I_Ba_* at the time point the *I–V* was calculated (average over 7–8 ms after depolarization); *P_max_* the maximum permeability; ν = *zFV*/(*RT*), with *z* being 2, *F* the Faraday constant, *R* the universal gas constant, *T* the absolute temperature, *V* the membrane potential. *[Ba]_i_* (set at 50 nM) and *[Ba]_o_* denote the intra- and extracellular Ba^2+^ concentration, respectively.

### Immunohistochemistry

Immunolabeling was performed on whole-mount organs of Corti of 4–6 week-old mice as described in Fell et al. (2016) using Zamboni’s fixative for 8 min on ice. Specimens were labeled using antibodies against Ca_v_1.3 (rabbit polyclonal, Alomone Labs, Israel, 1:500), BKα (rabbit polyclonal, Alomone Labs, Israel, 1:500; mouse monoclonal, antibodies-online, Germany, 1:500), GFP (goat polyclonal, Rockland, United States, 1:500), CtBP2/RIBEYE (mouse monoclonal, BD Transduction Laboratories, Germany, 1:100 – 1:200), SK2 (rabbit polyclonal, Sigma-Aldrich, Germany, 1:400), and calbindin (rabbit polyclonal, Swant Inc., Switzerland, 1:400). Primary antibodies were detected with Cy3-conjugated (donkey anti-rabbit: Jackson Immuno Research Laboratories, United Kingdom, 1:1500; donkey anti-goat: Abcam, United Kingdom, 1:1500) or Alexa 488-conjugated (anti-mouse: Invitrogen, United Kingdom, 1:500; anti-goat: Abcam, United Kingdom, 1:500) secondary antibodies. For immunolabeling experiments, at least three specimens of ≥3 animals were analyzed. *z*-stacks of fluorescence images were acquired using a confocal laser scanning microscope LSM710 (Carl Zeiss Microscopy GmbH, Germany). Images were analyzed with Fiji (Schindelin et al., 2012).

For quantification of Ca_v_1.3 clusters and RIBEYE-positive ribbons, images of 67.5 μm × 38.9 μm size covering eight IHCs were acquired at equal laser and gain settings, and maximum intensity projections (MIPs) were calculated. The channel of interest of a MIP image was background subtracted. A thresholded binary image was created (0 below threshold; 1 above threshold) with thresholds of 10% of the maximum intensity of the green color channel (RIBEYE) and 17% of the red color channel (Ca_v_1.3). Fluorescent dots < 0.05 μm^2^ were discarded. Size and number of clusters were analyzed using the particle count routine in Fiji and normalized to one IHC.

### Statistics

Data are provided as mean ± SD, unless otherwise stated. Depending on the distribution of the data, Ba^2+^ current properties, as well as size and number of Ca_v_1.3 clusters and ribbons were statistically analyzed using Student’s *t*-test or Mann–Whitney *U* test (*MWU* test; comparison of two groups) or using one-way *ANOVA* followed by Tukey *post hoc* test or Kruskal–Wallis test followed by Dunn-Holland-Wolfe *post hoc* test (comparison of > 2 groups) with Igor Pro software (WaveMetrics, United States) and SPSS statistics (IBM, Germany).

Statistical analysis of hearing measurements was performed with SPSS. Click ABR thresholds were analyzed using a one-way *ANOVA*, DPOAE amplitudes with a Kruskal–Wallis test and frequency-dependent ABR thresholds with a two-way *ANOVA*; all tests were followed by a Bonferroni *post hoc* test. ABR growth functions of amplitudes and latencies could not be tested by a two-way *ANOVA* due to unequal variances. Instead, a regression analysis was performed, and the parameters of the resulting regression lines (slope and *y*-axis intercept) were tested for differences using Student’s *t*-test or *MWU* test according to Sachs (1999).

## Results

### Cochlea-Specific Deletion of the Ca_v_1.3 Channel Using Ca_v_1.3-*flex* Mice With Cre Expression Under the *Pax2* Promoter

**FIGURE 1:**
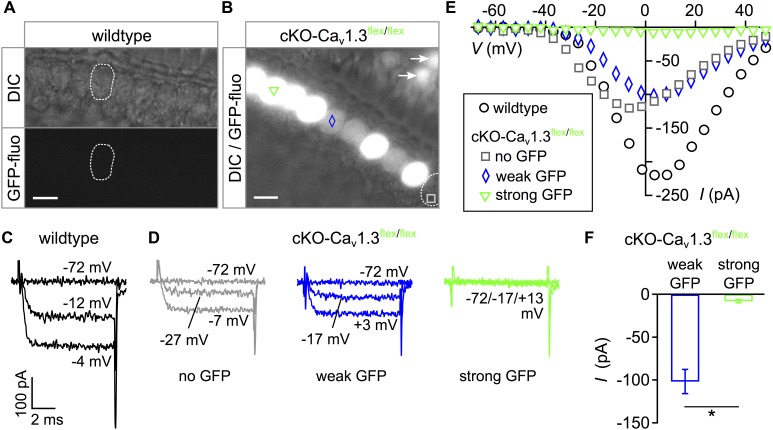
Intensity of eGFP fluorescence reflects success of Ca_v_1.3 ablation in cKO-Ca_v_1.3^flex/flex^ mice. **(A)** Apical turn IHCs of a whole-mount organ of Corti from a 3-week-old wildtype mouse did not show eGFP fluorescence. Top: DIC, bottom: GFP fluorescence. **(B)** Heterogeneous eGFP fluorescence (merged with DIC image) in IHCs of a 3-week-old cKO-Ca_v_1.3^flex/flex^ mouse with either strong (green triangle), weak (blue diamond) or no (gray square) fluorescence, respectively. Arrows: eGFP-positive OHCs. Scale bar in panels **(A,B)**: 10 μm. **(C,D)** Representative Ba^2+^ currents (*I*_Ba_) in response to 8-ms step depolarizations to the voltages indicated of a wildtype **(C)** and three cKO-Ca_v_1.3^flex/flex^ IHCs **(D)** with no (gray), weak (blue) or strong (green) eGFP fluorescence. **(E)** Corresponding individual *I*–*V* curves averaged during the last ms of the depolarizing step. **(F)** Peak *I*_Ba_ ± SD averaged from IHCs with weak (blue; *n* = 3) and strong GFP fluorescence (green; *n* = 4) of cKO-Ca_v_1.3^flex/flex^ mice (*MWU* test, ^∗^*P* = 0.0262).

In order to assess the phenotype of mice with cochlea-specific ablation of *Cacna1d* before birth, we analyzed GFP signals and whole-cell Ba^2+^ currents through Ca_v_1.3 channels in IHCs of conditional knockout (cKO) *Pax2::cre;Cacna1d-eGFP*^flex/flex^ mice, in short cKO-Ca_v_1.3^flex/flex^. GFP fluorescence of two distinct intensity levels was present in IHCs of the apical cochlear turn acutely dissected from 3-week-old cKO-Ca_v_1.3^flex/flex^ mice (Figure 1B) but not in wildtype IHCs (Figure 1A). Analysis of Ba^2+^ currents (*I*_Ba_) using 10 mM Ba^2+^ as a charge carrier in response to 8 ms step depolarizations revealed lack of *I*_Ba_ exclusively in those IHCs with a strong GFP signal (Figure 1D, green trace). In contrast, *I*_Ba_ was present in one IHC of cKO-Ca_v_1.3^flex/flex^ mice with weak (blue trace), one IHC without GFP fluorescence (gray trace, Figure 1D) and a wildtype IHC (Figure 1C). Corresponding individual peak *I–V* relations show that *I*_Ba_ was abolished in the IHC with strong GFP fluorescence whereas it was present in the two cKO-Ca_v_1.3^flex/flex^ IHCs with weak or no GFP fluorescence and in the wildtype IHC (Figure 1E). Averaged peak *I*_Ba_ from cKO-Ca_v_1.3^flex/flex^ IHCs with strong GFP (–7.9 ± 1.9 pA; *n* = 4) was significantly reduced compared with IHCs showing weak GFP fluorescence (–101.6 ± 14.2 pA; *n* = 3; *P* = 0.0262, *MWU* test, Figure 1F). We concluded that only those IHCs with strong fluorescence represented true knockout cells with two switched *flex* alleles whereas IHCs with weak fluorescence represented cells with one switched and one intact *flex* allele.

**FIGURE 2:**
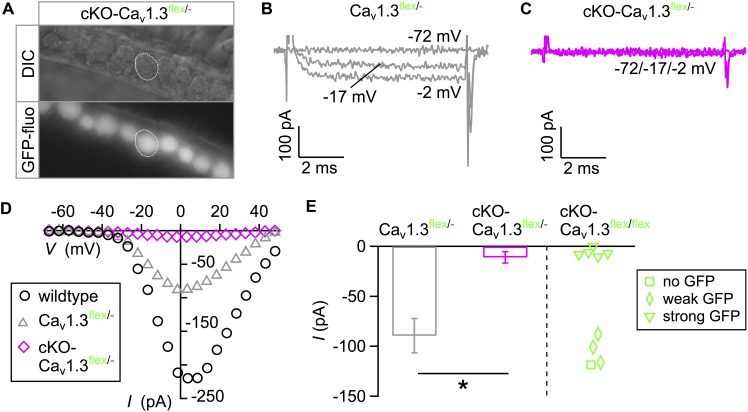
eGFP fluorescence faithfully reflects *I*_Ba_ ablation in IHCs of cKO-Ca_v_1.3^flex/-^ mice. **(A)** Uniform eGFP fluorescence (bottom) in IHCs (top: DIC image) from an apical cochlear turn of a 3-week-old cKO-Ca_v_1.3^flex/-^ mouse. **(B,C)** Representative *I*_Ba_ responses to 8-ms step depolarizations of a Ca_v_1.3^flex/-^ control **(B)** and an eGFP-positive cKO-Ca_v_1.3^flex/-^ IHC **(C)** to the voltages indicated. **(D)** *I*–*V* curves corresponding to panels **(B)** and **(C)** and a wildtype IHC (Figure 1C). **(E)** Left: Average peak *I*_Ba_ ± SD of 10 Ca_v_1.3^flex/-^ and 15 cKO-Ca_v_1.3^flex/-^ IHCs (*MWU* test, ^∗∗∗^*P* < 0.001). Right: *I*_Ba_ of individual IHCs with no (square; *n* = 1), weak (diamonds; *n* = 3) or strong (triangles; *n* = 5) eGFP fluorescence from cKO-Ca_v_1.3^flex/flex^ mice.

In order to increase the ratio of “true knockout” IHCs without remaining intact *flex* alleles, cKO-Ca_v_1.3^flex/flex^ mice were crossbred with Ca_v_1.3^-/-^ mice resulting in cKO-Ca_v_1.3^flex/-^ mice, in which Cre needed to cut and switch only one *flex* allele per cell. Here, GFP fluorescence was uniform in IHCs of acutely dissected organs of Corti from 3-week-old cKO-Ca_v_1.3^flex/-^ mice (Figure 2A). Typical *I*_Ba_ traces (Figure 2B,C) and corresponding *I–V* curves (Figure 2D) show that *I*_Ba_ was abolished in GFP-positive IHCs of cKO-Ca_v_1.3^flex/-^ mice (Figure 2C) but retained in control IHCs of Ca_v_1.3^flex/-^ mice (Figure 2B) where the *flex* allele was not switched due to absence of Cre. *I–V* curves further showed a reduction of *I*_Ba_ in control Ca_v_1.3^flex/-^ (gray) compared with wildtype IHCs (black, Figure 2D). The average peak *I*_Ba_ from cKO-Ca_v_1.3^flex/-^ IHCs was significantly reduced compared with control Ca_v_1.3^flex/-^ IHCs (Figure 2E, bars; *MWU* test, *P* < 0.001). Peak *I*_Ba_ from individual cKO-Ca_v_1.3^flex/flex^ IHCs demonstrate that the *I*_Ba_ amplitude of IHCs with strong GFP fluorescence (cf. Figure 1) resembled that of GFP-positive IHCs of cKO-Ca_v_1.3^flex/-^ mice whereas *I*_Ba_ values of IHCs with weak or no GFP fluorescence were similar to those of Ca_v_1.3^flex/-^ IHCs (Figure 2E, right). The lack of *I*_Ba_ was accompanied by a reduction in cell size as evident by a significantly reduced membrane capacitance (*P* < 0.001, Kruskal–Wallis Test) in cKO-Ca_v_1.3*^flex/-^* mice (6.7 ± 0.6 pF; *n* = 15; *P* = 0.001, effect of genotype) but not in control Ca_v_1.3*^flex/-^* mice (9.5 ± 1.4 pF; *n* = 10) compared with the wildtype (8.7 ± 1.0 pF; *n* = 10).

In summary, heterogeneous GFP expression and persistence of *I*_Ba_ in IHCs with weak GFP fluorescence of cKO-Ca_v_1.3^flex/flex^ mice show that (i) Cre did not faithfully switch both *flex* alleles at 3 weeks of age and (ii) GFP fluorescence is no reliable marker for deletion of Ca_v_1.3 channels in IHCs of cKO-Ca_v_1.3^flex/flex^ mice. In contrast, in IHCs of cKO-Ca_v_1.3*^flex/-^* mice containing only one *flex* allele, GFP fluorescence unequivocally indicated a cellular knockout genotype.

### GFP Toxicity in IHCs of cKO-Ca_v_1.3^flex/flex^ Mice

**FIGURE 3:**
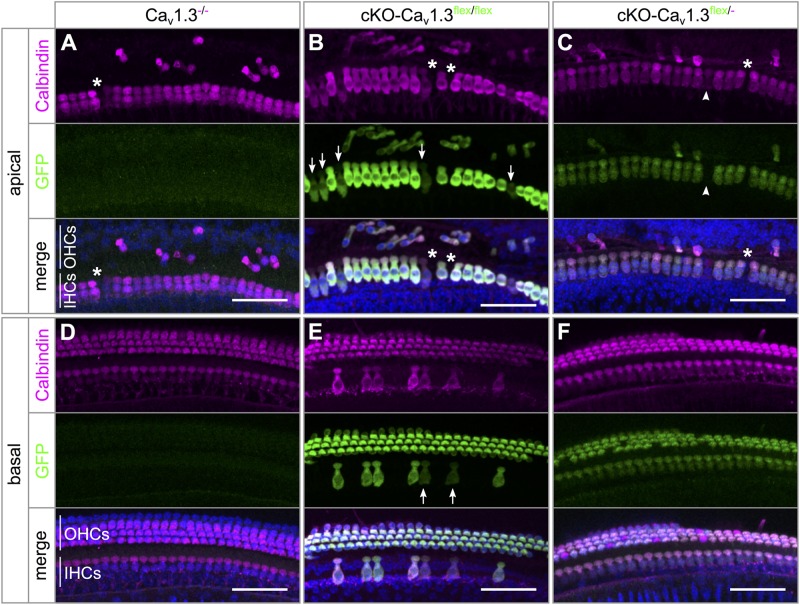
Pattern of eGFP immunolabeling and degree of basal-turn degeneration differ between IHCs of cKO-Ca_v_1.3^flex/flex^ and cKO-Ca_v_1.3^flex/-^ mice. Maximum intensity projections (MIP) of confocal stacks of whole-mount organs of Corti from 4 to 5 week-old mice. Stretches of ∼25 IHCs and adjacent OHCs from apical **(A–C)** and basal **(D–F)** cochlear turns of a Ca_v_1.3^-/-^ **(A,D)**, a cKO-Ca_v_1.3^flex/flex^ **(B,E)** and a cKO-Ca_v_1.3^flex/-^ mouse **(C,F)** were co-immunolabeled for the hair cell marker calbindin (magenta, top) and for GFP (green, middle). Merged colors are shown below with DAPI-stained nuclei (blue). **(A–C)** GFP labeling was irregular among IHCs of a cKO-Ca_v_1.3^flex/flex^ mouse (arrows: IHCs with weak GFP labeling), but regular among IHCs of a cKO-Ca_v_1.3^flex/-^ mouse (arrowhead: IHC without GFP signal). Independently of the genotype, Ca_v_1.3-deficiency in the apical cochlear turn resulted in degeneration of few IHCs [asterisks in panels **(A–C)**], whereas the majority of OHCs were degenerated. **(D)** No degeneration of IHCs or OHCs in the basal cochlear turn of a Ca_v_1.3^-/-^ mouse. **(E)** In a cKO-Ca_v_1.3^flex/flex^ mouse, the majority of IHCs was degenerated, with the remaining IHCs showing heterogeneous GFP labeling (arrows: weak GFP signal). **(F)** Uniform GFP labeling in IHCs and lack of hair cell degeneration in the basal turn of a cKO-Ca_v_1.3^flex/-^ mouse. Scale bar: 50 μm.

In Ca_v_1.3^-/-^ mice, mild degeneration of IHCs has been reported in the apical cochlear turn starting after P20 and in the basal cochlear turn after P35 (Platzer et al., 2000; Glueckert et al., 2003; Nemzou et al., 2006). Degeneration of IHCs after cochlea-specific deletion of Ca_v_1.3 was analyzed in organs of Corti of 4–5 week-old cKO-Ca_v_1.3^flex/flex^ and cKO-Ca_v_1.3*^flex/-^* compared with Ca_v_1.3^-/-^ mice, which were double-immunolabeled for GFP and the hair-cell marker calbindin (Figure 3 and Table 1).

**Table 1:** Degeneration of IHCs in the apical and basal cochlear turn of mice with systemic (Ca_v_1.3^-/-^) and cochlea-specific deletion of Ca_v_1.3 with GFP reporter function (cKO-Ca_v_1.3^flex/flex^ and cKO-Ca_v_1.3^flex/-^).

	Ca_v_1.3^-/-^		cKO-Ca_v_1.3^flex/flex^		cKO-Ca_v_1.3^flex/-^	
			
	Apical	Basal	Apical	Basal^∗^	Apical	Basal
*n* ears/animals	4/3	4/3	8/4	5/4	4/4	3/3
IHCs / ear	107.3 ± 56.4	59.5 ± 29.5	156.5 ± 61.4	10.0 ± 8.2	164.8 ± 82.2	44.3 ± 38.8
Degenerated IHCs / ear	7.25 ± 5.3	0.3 ± 0.5	8.5 ± 10.2	44.8 ± 28.8	1.8 ± 1.0	0.0 ± 0.0
Degeneration (%) / ear	9.5 ± 10.2%	0.5 ± 0.9%	6.5 ± 9.1%	83.1 ± 4.7%	1.2 ± 0.7%	0.0 ± 0.0%
IHC slots, total	458	239	1320	274	666	131
IHCs, total	429	238	1252	50	659	131
Degenerated IHCs, total	29	1	68	224	7	0
Degeneration (%), total	6.3%	0.4%	6.2%	81.8%	1.1%	0%


*The degree of degeneration was determined for individual ears and cochlear location (apical vs. basal) and is given as number of empty IHC slots (degenerated IHCs) per number of total IHC slots (filled + empty). Numbers per ear are given as mean ± SD. ^∗^due to profound degeneration, degenerated IHCs were determined as OHCs of the innermost OHC row minus intact IHCs.*

IHCs of all three genotypes showed mild IHC loss of ≤6.3% in the apical turn (Figure 3A–C and Table 1). In contrast, the majority (81.3%) of basal-turn IHCs of cKO-Ca_v_1.3^flex/flex^ mice was missing (Figure 3E and Table 1). This pronounced degeneration was not caused by lack of Ca_v_1.3 because basal-turn IHCs of Ca_v_1.3^-/-^ and cKO-Ca_v_1.3^flex/-^ mice did not show any degeneration (<0.5%; Figure 3D,F and Table 1). We conclude that high expression levels of GFP caused by two functional *flex* alleles in cKO-Ca_v_1.3^flex/flex^ mice (cf. Figure 1A) resulted in a toxic effect of GFP on basal IHCs. The lack of IHC degeneration in the basal cochlea from cKO-Ca_v_1.3^flex/-^ mice suggests a dose-dependent toxicity of GFP that requires more than one functional GFP allele.

The majority of outer hair cells (OHCs) from the apical (Figure 3A–C) but not basal cochlear turn (Figure 3D–F) were degenerated, as described before for Ca_v_1.3^-/-^ mice (Platzer et al., 2000; Glueckert et al., 2003; Engel et al., 2006). Thus, cochlea-specific deletion of Ca_v_1.3 channels coupled to GFP expression resulted in a similar degeneration of apical turn OHCs as observed in systemic Ca_v_1.3^-/-^ mice.

The patterns of IHC GFP labeling in either cKO-Ca_v_1.3^flex/flex^ (Figure 3B,E) or cKO-Ca_v_1.3^flex/-^ mice (Figure 3C,F) were similar to the eGFP fluorescence patterns in acutely dissected organs of Corti from these genotypes (Figure 1A, 2A). GFP labeling intensity was heterogeneous between individual IHCs of cKO-Ca_v_1.3^flex/flex^ mice with either intense or weak (arrows) labeling (Figure 3B,E) but uniform in IHCs of cKO-Ca_v_1.3^flex/-^ mice (Figure 3C,F), with few individual IHCs not being labeled (Figure 3C, arrowheads).

The rate of true knockout IHCs was assessed by quantification of apical-turn organs of Corti immunolabeled for GFP, Ca_v_1.3 and/or BK channels (Table 2). In cKO-Ca_v_1.3^flex/-^ mice, the knockout rate was 89.2%, which was only slightly higher than the knockout rate of 87.4% in cKO-Ca_v_1.3^flex/flex^ mice at 4–5 weeks of age. Although replacing one *flex* allele by a knockout (–) allele increased the success rate of Cre in switching one *flex* allele at 3 weeks of age (cf. Figure 1A,B, 2A), Cre caught up in switching both *flex* alleles in cKO-Ca_v_1.3^flex/flex^ mice 2 weeks later.

**Table 2:** Rate of successful *flex* switch in IHCs from the apical cochlear turn of cKO-Ca_v_1.3^flex/flex^ and cKO-Ca_v_1.3^flex/-^mice at 4–6 weeks of age.

	cKO-Ca_v_1.3^flex/flex^		cKO-Ca_v_1.3^flex/-^	
		
	Mean ± SD per animal (*n* = 3)	% of IHC slots	Mean ± SD per animal (*n* = 5)	% of IHC slots
Total IHC slots (filled and empty)	285.7 ± 103.7	100.0	203.4 ± 95.2	100.0
Heterozygous IHCs	36.0 ± 36.5	12.6	22.0 ± 15.4	10.8
Knockout IHCs	207.0 ± 104.5	72.5	181.4 ± 90.5	89.2
Degenerated IHCs	42.7 ± 61.0	14.9	0.0 ± 0.0	0
cKO-IHCs	249.7 ± 67.9	87.4	181.4 ± 90.5	89.2


*Organs of Corti labeled for Ca_v_1.3, BK and/or GFP were divided in four different groups according to their phenotype: (i) knockout IHCs: lack of Ca_v_1.3- or BK-positive labeling (cf. Figure 5, 6) or strong GFP labeling in cKO-Ca_v_1.3^flex/flex^ or GFP labeling in cKO-Ca_v_1.3^flex/-^ mice, respectively; (ii) degenerated IHCs: empty IHC-sized slots; (iii) heterozygous IHCs: Ca_v_1.3- or BK-positive labeling or weak GFP labeling in cKO-Ca_v_1.3^flex/flex^ or lack of GFP labeling in cKO-Ca_v_1.3^flex/-^ mice, respectively. Wildtype-like IHCs lacking GFP labeling were not found in cKO-Ca_v_1.3^flex/flex^ mice. Assuming that only knockout IHCs degenerated, the percentage of IHCs with a successful deletion of Ca_v_1.3 channels was calculated from the sum of knockout and degenerated IHCs (cKO-IHCs).*

In conclusion, the percentage of knockout IHCs finally was not increased by replacement of one *flex* allele with a knockout (–) allele to obtain cKO-Ca_v_1.3^flex/-^ mice. However, we found that in cKO-Ca_v_1.3^flex/flex^ mice (i) basal-turn IHCs degenerated, most likely due to dose-dependent GFP toxicity and (ii) GFP expression in IHCs was not unequivocally associated with deletion of Ca_v_1.3 channels, thus leading us to further use cKO-Ca_v_1.3^flex/-^ instead of cKO-Ca_v_1.3^flex/flex^ mice.

### Similar Phenotypes of IHCs From cKO-Ca_v_1.3^flex/-^ and Systemic Ca_v_1.3^-/-^ Mice

**FIGURE 4:**
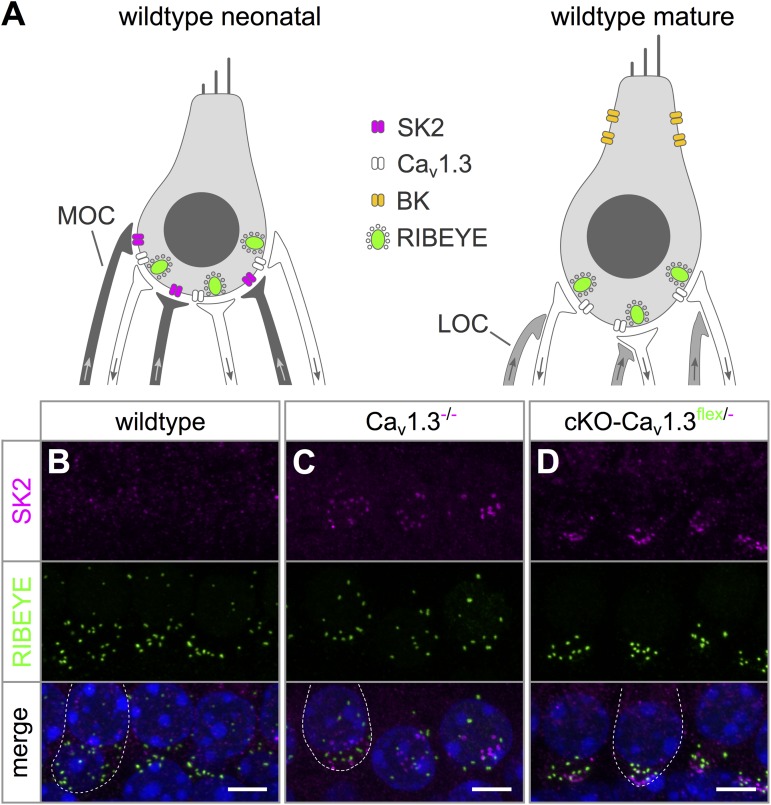
Persistence of SK2 channels in IHCs of cKO-Ca_v_1.3^flex/-^ mice. **(A)** Sketch of a wildtype IHC before (left, neonatal) and after the onset of hearing (right, mature) depicting differences in shape, ion channel composition and innervation. Immature IHCs express SK2 channels (magenta) at the basal pole, which are down-regulated after the onset of hearing, and do not possess BK channels. At the onset of hearing, BK channels are up-regulated and localize mainly to the neck of mature IHCs. Medial olivocochlear (MOC) efferent fibers of the SOC (dark gray) innervate IHCs of neonatal mice. Whereas mature IHCs lack direct efferent innervation, their afferent fibers receive lateral olivocochlear (LOC) efferent fibers (light gray). **(B)** SK2 labeling (top, magenta) was absent from apical-turn wildtype IHCs at 4–6 weeks of age. **(C,D)** Dot-like SK2 labeling was present at the basal pole, but not co-localized with synaptic ribbons (RIBEYE, middle, green) of apical-turn IHCs of a Ca_v_1.3^-/-^**(C)** and a cKO-Ca_v_1.3^flex/-^ mouse at the age of 4–6 weeks. The merged image is shown with nuclei stained in blue with DAPI. Scale bars: 5 μm.

In wildtype mice, up-regulation of BK K^+^ channels around the onset of hearing (P12) and down-regulation of neonatal SK2 K^+^ channels mark the end of terminal maturation and the onset of the mature function of IHCs (Figure 4A) (Kros et al., 1998; Marcotti et al., 2004). In systemic Ca_v_1.3^-/-^ mice, IHCs maintain an immature-like ion channel composition with persistent expression of SK2 but lack of BK K^+^ channels (Brandt et al., 2003; Engel et al., 2006; Nemzou et al., 2006). The failure of acquiring a mature composition of K^+^ channels may have been caused by lack of Ca_v_1.3 currents (i) in the IHC itself or (ii) in brainstem nuclei causing an altered efferent input on the IHC (Hirtz et al., 2011, 2012; Satheesh et al., 2012). Precise timing and patterning of Ca^2+^ action potentials generated by IHCs during a critical period before the onset of hearing are crucial for their maturation (Johnson et al., 2013a). Altered neuronal activity of the efferent input onto neonatal IHCs therefore might also affect their Ca^2+^ action potentials and hence their development.

SK2 immunolabeling was localized at the basolateral pole apart from synaptic ribbons (RIBEYE) of apical turn IHCs from 4 to 5 week-old cKO-Ca_v_1.3^flex/-^ and Ca_v_1.3^-/-^ mice (Figure 4C,D) indicating an immature phenotype. In contrast, no SK2 labeling was found at the basolateral pole of wildtype IHCs (Figure 4B).

**FIGURE 5:**
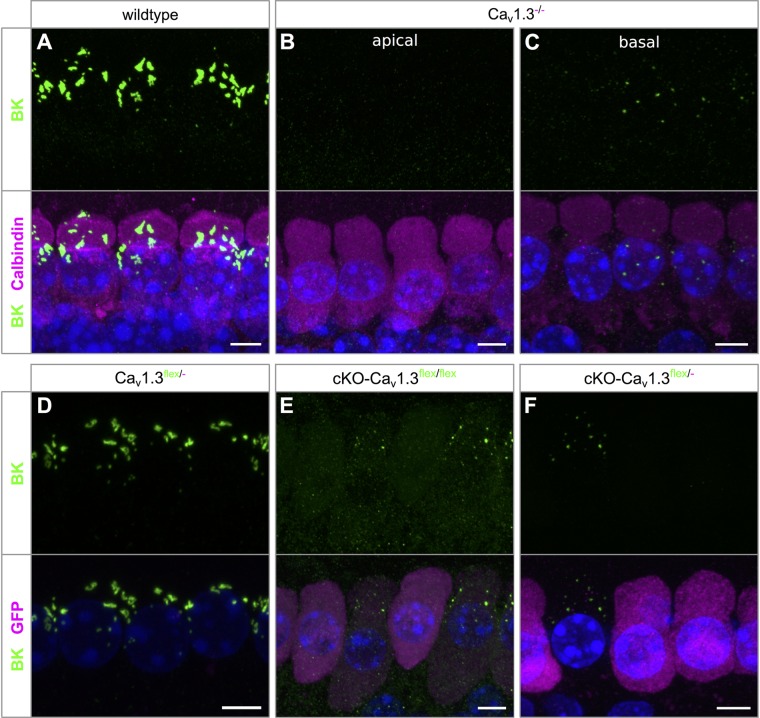
BK channel expression is related to reduced GFP labeling in cKO-Ca_v_1.3^flex/flex^ and cKO-Ca_v_1.3^flex/-^ IHCs. **(A–C)** MIP of confocal stacks of IHCs labeled for BK (green) and calbindin (magenta) of a wildtype **(A)** and a Ca_v_1.3^-/-^ mouse **(B,C)** at the age of 4–5 weeks. **(A)** Large spots of BK immunoreactivity at the neck of wildtype IHCs. **(B,C)** In Ca_v_1.3^-/-^ IHCs, BK immunolabeling was absent from apical-turn IHCs **(B)** and vastly reduced in IHCs of the basal turn **(C)** compared with apical-turn wildtype IHCs **(A)**. **(D–F)** Co-labeling for BK channels (green) and GFP (magenta) in apical-turn IHCs of a control Ca_v_1.3^flex/-^ **(D)**, a cKO-Ca_v_1.3^flex/flex^ **(E)** and a cKO-Ca_v_1.3^flex/-^ mouse **(F)**. **(D)** Normal, wildtype-like BK labeling at the neck in control Ca_v_1.3^flex/-^ IHCs. **(E,F)** BK labeling was absent from those IHCs of the cKO-Ca_v_1.3^flex/flex^ mouse with strong GFP labeling **(E)** and from GFP-positive IHCs of the cKO-Ca_v_1.3^flex/-^ mouse **(F)**. Note dot-like BK labeling in those IHCs of the cKO-Ca_v_1.3^flex/flex^ mouse with weak GFP expression **(E)** and in a GFP-negative IHC of the cKO-Ca_v_1.3^flex/-^ mouse **(F)**, both indicative of a non-switched *flex* allele. Scale bar: 5 μm.

BK channel expression was assessed in wildtype and Ca_v_1.3^-/-^ IHCs co-labeled with calbindin (Figure 5A–C) and in Ca_v_1.3^flex/-^ controls, cKO-Ca_v_1.3^flex/flex^ and cKO-Ca_v_1.3^flex/-^ IHCs co-labeled with GFP (Figure 5D–F). BK channels, which are indicators of a mature IHC phenotype, were present at the neck of wildtype IHCs (Figure 5A). In Ca_v_1.3^-/-^ mice, BK labeling was absent from apical-turn IHCs (Figure 5B), whereas sparse and faint labeling was found in basal-turn IHCs (Figure 5C). In Ca_v_1.3^flex/-^ control IHCs, normal BK labeling was found at the neck of IHCs (Figure 5D). In true cKO IHCs i.e., IHCs with strong GFP labeling in cKO-Ca_v_1.3^flex/flex^ mice (Figure 5E) and with GFP labeling in cKO-Ca_v_1.3^flex/-^ mice (Figure 5F), BK labeling was missing. Unexpectedly, BK immunolabeling in heterozygous IHCs of both cKO genotypes, i.e., IHCs with weak (cKO-Ca_v_1.3^flex/flex^, Figure 5E) or no GFP labeling (cKO-Ca_v_1.3^flex/-^, Figure 5F), which appeared as small dots at the neck, clearly differed from the large BK patches in control Ca_v_1.3^flex/-^ (Figure 5D) or wildtype IHCs (Figure 5A). The GFP-negative IHCs of cKO-Ca_v_1.3^flex/-^ mice containing a non-switched *flex* allele should, however, have the same phenotype and thus the same BK labeling pattern as IHCs of control Ca_v_1.3^flex/-^ mice.

### Reduced Ba^2+^ Currents and Ca_v_1.3 Protein Clusters in Control Ca_v_1.3^flex/-^ IHCs

**FIGURE 6:**
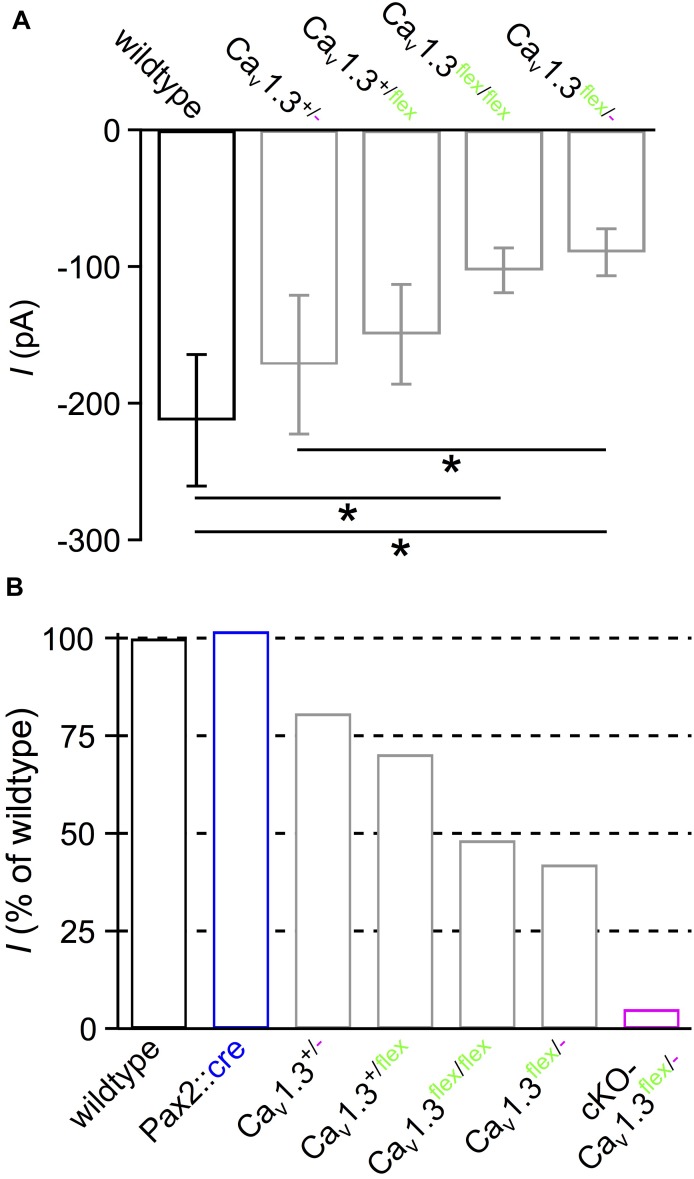
Reduced *I*_Ba_ amplitude in IHCs with unswitched *flex* alleles. **(A)** Averaged peak *I*_Ba_ ± SD of 10 wildtype, 10 Ca_v_1.3^+/-^, 7 Ca_v_1.3^+/flex^, 10 Ca_v_1.3^flex/flex^ and 10 Ca_v_1.3^flex/-^ IHCs reveal that *I*_Ba_ was reduced when at least one wildtype allele was replaced by a *flex* allele (^∗^ effect of genotype; *P* < 0.001, Kruskal–Wallis Test). **(B)** Percentage of average peak *I*_Ba_ of IHC groups from panel **(A)**, 10 *Pax2::cre* controls and 15 cKO-Ca_v_1.3^flex/-^ IHCs (Figure 2E), all normalized to the wildtype (100%).

We noticed that expression of one or two *flex* alleles without Cre resulted in smaller IHC Ba^2+^ currents compared with wildtype IHCs (cf. Figure 1D,E, 2B–E). However, in conditional mouse lines, the function of the target gene should remain unaltered unless it is deleted or manipulated by Cre or other recombinases. For generating the conditional *Cacna1d* construct, *loxP* sites were inserted in intronic regions flanking exon 2 of the *Cacna1d* gene, which should not impair its function (Satheesh et al., 2012). To determine the side effect of the construct in the *Cacna1d-eGFP^flex^* allele, we measured *I*_Ba_ in IHCs of mice with different combinations of wildtype (+), *flex* and knockout (-) alleles, i.e., Ca_v_1.3^+/-^, Ca_v_1.3*^+/flex^*, Ca_v_1.3^flex/flex^ and Ca_v_1.3^flex/-^ compared with wildtype mice (Figure 6). Averaged peak *I*_Ba_ amplitudes of IHCs from Ca_v_1.3^flex/flex^ (–102.7 ± 16.4 pA; *n* = 10) and Ca_v_1.3^flex/-^ mice (–89.5 ± 17.2 pA; *n* = 10) were significantly reduced compared with wildtype (–212.4 ± 48.2 pA; *n* = 10; *P* < 0.001, Kruskal–Wallis Test; Figure 6A). *I*_Ba_ normalized to the wildtype (100%) was reduced to 48.4% in Ca_v_1.3^flex/flex^ and 42.1% in Ca_v_1.3^flex/-^ IHCs, respectively (Figure 6B). In mice with only one wildtype (+) allele, *I*_Ba_ was slightly but not significantly reduced to –171.6 ± 50.9 pA or 80.9% (Ca_v_1.3^+/-^; *n* = 10) and –149.5 ± 36.5 pA or 70.4% (Ca_v_1.3^+/*flex*^; *n* = 7; Figure 6A,B), respectively. In contrast, *I*_Ba_ was reduced to 5.1% in IHCs of cKO-Ca_v_1.3^flex/-^ mice indicating a complete loss of Ca_v_1.3 channels leaving a small residual Ca^2+^ current that has been described before in the systemic knockout (Platzer et al., 2000; Brandt et al., 2003; Dou et al., 2004). Additionally, Cre expression in the cochlea did not affect *I*_Ba_ in IHCs of *Pax2::cre* control mice (–216.4 ± 53.1 pA; *n* = 10; Figure 6B). Analysis of gating properties by fitting the *I–V* curves to a second-order Boltzmann function times Goldman-Hodgkin-Katz driving force yielded a small but significant shift of *V_h_* by –2.5 mV in Ca_v_1.3^flex/-^ (–12.6 ± 2.0 mV; *n* = 10) versus wildtype IHCs (–10.1 ± 2.3 mV; *n* = 10; *P* = 0.019, *MWU* test), whereas the voltage sensitivity of activation determined by the slope factor *k* was unaffected (Ca_v_1.3^flex/-^: 11.22 ± 0.97 mV; wildtype: 11.26 ± 0.30 mV; *P* = 0.762, *MWU* test).

In summary, reduction of *I*_Ba_ amplitude in IHCs of control Ca_v_1.3^flex/flex^ and Ca_v_1.3^flex/-^ mice, as well as altered gating properties in Ca_v_1.3^flex/-^ control IHCs demonstrate that the unswitched *Cacna1d flex* allele functionally does not fully replace the wildtype allele.

**FIGURE 7:**
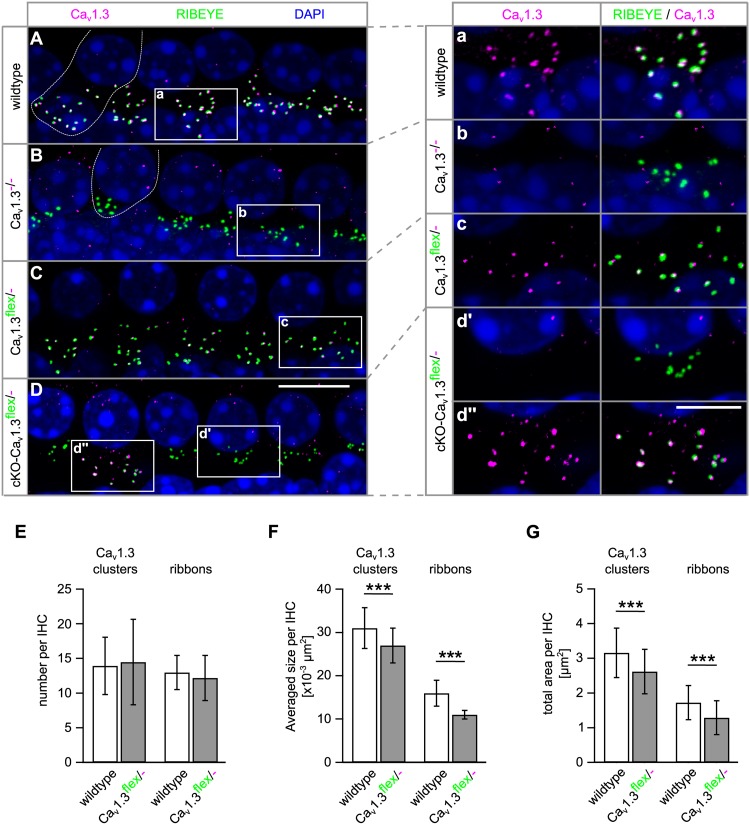
Presynaptic Ca_v_1.3 clusters are missing at the IHC synapse of cKO-Ca_v_1.3^flex/-^ mice and are smaller at IHC synapses of Ca_v_1.3^flex/-^ control mice. **(A–D)** MIP of confocal stacks of whole-mount preparations of apical turn organs of Corti co-immunolabeled for Ca_v_1.3 and RIBEYE at 4 weeks of age. Stretches with four to five IHCs of a wildtype **(A)**, a Ca_v_1.3^-/-^ **(B)**, a Ca_v_1.3^flex/-^ control **(C)** and a cKO-Ca_v_1.3^flex/-^ mouse **(D)** labeled with anti-Ca_v_1.3 (magenta) and anti- RIBEYE (green) are shown, with one wildtype and one Ca_v_1.3^-/-^ IHC indicated by a dashed line in panels **(A)** and **(B)**. **(a–d″)** Enlarged synaptic poles of IHCs from boxes in panels **(A–D)** shown with split color channels and as merge. **(E–G)** Quantitative analysis of the Ca_v_1.3 clusters and RIBEYE-labeled ribbons in IHCs of Ca_v_1.3^flex/-^ control and wildtype mice. **(E)** Number of Ca_v_1.3 clusters (*P* = 0.69) and ribbons per IHC (*P* = 0.074) were unaffected in Ca_v_1.3^flex/-^ control mice (*MWU* test). **(F)** Average sizes per IHC of both, Ca_v_1.3 clusters and ribbons were reduced in Ca_v_1.3^flex/-^ control mice (^∗∗∗^*P* < 0.001, *MWU* test). **(G)** This reduction also applied to the total area of Ca_v_1.3 clusters and of ribbons per IHC in Ca_v_1.3^flex/-^ control mice (^∗∗∗^*P* < 0.001, Student’s *t*-test). Number of ears/animals/images analyzed (with eight IHCs per image), respectively: wildtype, 4/2/56; Ca_v_1.3^flex/-^ control mice: 5/3/100; scale bars: **(A–D)**, 10 μm; **(a–d″)**, 5 μm.

The functional reduction of Ca_v_1.3 channels might be caused by a reduced amount of Ca_v_1.3 channel protein in the IHC membrane or by a reduced function of Ca_v_1.3 channels in Ca_v_1.3^flex/-^ mice. The abundance of Ca_v_1.3 channel protein was assessed by co-immunolabeling for Ca_v_1.3 (magenta) and synaptic ribbons (RIBEYE, green, Figure 7). Ca_v_1.3 clusters were localized at the synaptic ribbons of wildtype IHCs (Figure 7A,a) and at the majority of ribbons of IHCs from Ca_v_1.3^flex/-^ control mice (Figure 7C,c). In contrast, no specific Ca_v_1.3 labeling was found at the synapses of Ca_v_1.3^-/-^ IHCs (Figure 7B,b) and most, but not all IHCs of cKO-Ca_v_1.3^flex/-^ mice (Figure 7D,d′). In part of the IHCs from cKO-Ca_v_1.3^flex/-^ mice, Ca_v_1.3 labeling was still present at the synaptic ribbons (Figure 7D,d″) indicating that the *flex* allele was not switched in these cells. Synaptic ribbons (RIBEYE) of Ca_v_1.3-deficient IHCs from Ca_v_1.3^-/-^ and cKO-Ca_v_1.3^flex/-^ mice were agglomerated and localized closer to the nucleus (Figure 7B,D) as described before (Nemzou et al., 2006).

To elucidate the cause of the reduced *I*_Ba_ amplitude (42% of wildtype, Figure 6A,B) in IHCs of Ca_v_1.3^flex/-^ control mice, a quantitative analysis of the size and number of Ca_v_1.3 clusters and synaptic ribbons was performed (Figure 7E–G). Whereas the number of Ca_v_1.3 clusters and ribbons was unchanged, the average size of both Ca_v_1.3 clusters and synaptic ribbons was significantly reduced to 73 and 89% in Ca_v_1.3^flex/-^ control IHCs compared with wildtype (Figure 7F). This reduction in size also applied to the total area of Ca_v_1.3 clusters to 75% and of ribbons to 83% of the total areas in wildtype, respectively (Figure 7G). In conclusion, less Ca_v_1.3 protein was produced in IHCs of Ca_v_1.3^flex/-^ mice evident by reduced *I_Ba_* amplitudes and smaller Ca_v_1.3 channel clusters, which was accompanied by smaller ribbons.

### Profound Hearing Loss in cKO-Ca_v_1.3^flex/-^ and Mild Hearing Impairment in Control Ca_v_1.3^flex/-^ Mice

**FIGURE 8:**
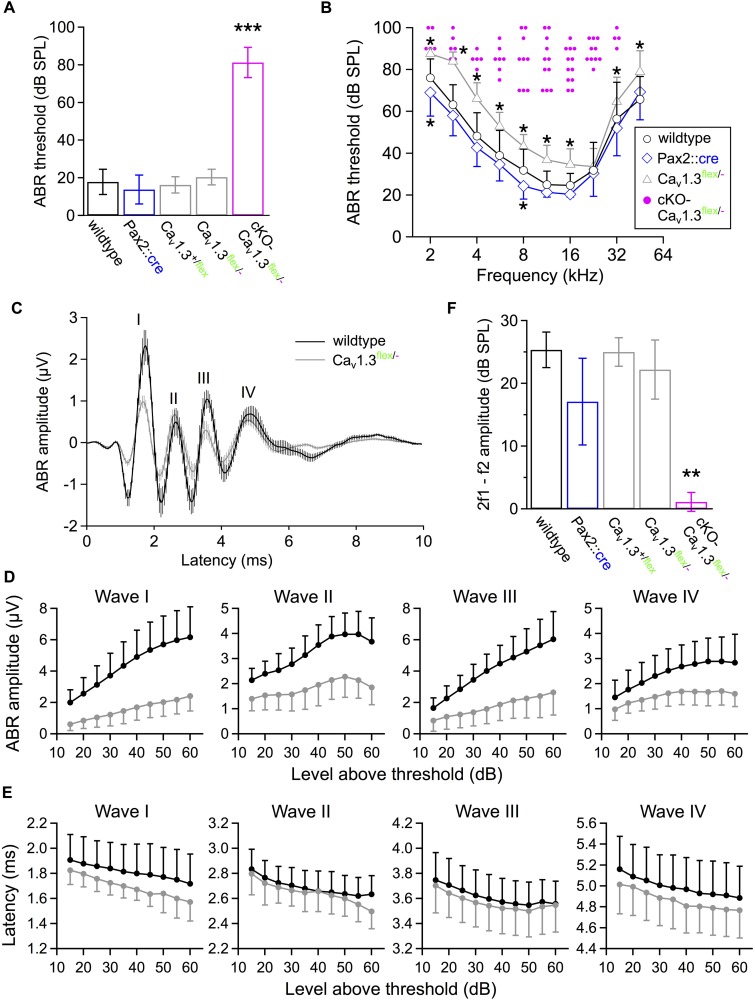
Hearing function of 4–6 week-old cKO-Ca_v_1.3^flex/-^ and control mice assessed by measurements of auditory brainstem responses (ABR) and distortion product otoacoustic emissions (DPOAE). **(A)** Click-evoked ABR thresholds (mean ± SD) were measured when ≤100 dB SPL in 12/7 out of 16/8 ears/animals of cKO-Ca_v_1.3^flex/-^ mice. They were significantly increased compared with wildtype (14/7 ears/animals) and controls (*Pax2::cre*, 16/8 ears/animals; Ca_v_1.3^+/flex^, 12/6 ears/animals; Ca_v_1.3^flex/-^, 14/7 ears/animals, ^∗∗∗^*P <* 0.001, one-way ANOVA with Bonferroni *post hoc* test). **(B)** Frequency-specific ABR thresholds of cKO-Ca_v_1.3^flex/-^ mice (single data, pink) could be determined in ears/animals: 2 kHz, 5/3; 2.8 kHz, 6/3; 4 kHz, 6/3; 5.6 kHz, 7/4; 8 kHz, 10/5; 11.3 kHz, 12/7; 16 kHz, 12/6; 22.6 kHz, 10/6; 32 kHz, 4/4; 45.2 kHz, 0/0; out of a total of 16/8 ears/animals recorded. Frequency-dependent ABR thresholds were increased in Ca_v_1.3^flex/-^ control mice (14/7 ears/animals; group effect of two-way ANOVA with Bonferroni *post hoc* test, *P* < 0.001; effect of genotype at 2, 2.8, 4, 5.6, 8, 11.3, 16, 32 and 45.2 kHz) and reduced in *Pax2::cre* control mice (15/8 ears/animals; group effect of two-way ANOVA with Bonferroni *post hoc* test, *P* < 0.001; ^∗^ effect of genotype at 2 and 8 kHz) compared with wildtype (14/7 ears/animals). **(C)** Averaged ABR waveforms to click stimuli at 40 dB above threshold (mean ± SEM) for 14/7 wildtype ears/animals (black) and 14/7 Ca_v_1.3^flex/-^ control ears/animals (gray). **(D)** Mean DPOAE maximum amplitudes ± SD with f1 starting at 10.0 kHz, L1 = 55 dB SPL, f2 = 1.2 × f1 averaged over 10–18 kHz in 0.5 kHz steps, and L2 = 45 dB SPL were reduced in cKO-Ca_v_1.3^flex/-^ mice (14/7 ears/animals) compared with wildtype (14/7 ears/animals) and controls (*Pax2::cre*, 16/8 ears/animals; Ca_v_1.3^+/flex^, 12/6 ears/animals; Ca_v_1.3^flex/-^, 14/7 ears/animals; Kruskal–Wallis with Bonferroni *post hoc* test, ^∗∗^*P ≤* 0.008). **(E,F)** Growth functions of amplitudes and latencies of waves I to IV (mean ± SD) revealed reduced mean amplitude values for all four waves and all stimulus levels in Ca_v_1.3^flex/-^ control (gray; *n* = 14/7 ears/animal) compared with wildtype mice (black; *n* = 12–14/6–7 ears/animals). For clarity, the SD is plotted in one direction only (+SD or -SD).

Next, we assessed how the loss/reduction of Ca_v_1.3 channels affected the auditory function of cKO-Ca_v_1.3^flex/-^ and control mice. In 4–6 week-old cKO-Ca_v_1.3^flex/-^ mice, click-evoked ABR thresholds (Figure 8A) were absent (threshold > 100 dB SPL) in 4/3 ears/animals and significantly elevated in the remaining 12/7 out of 16/8 ears/animals (81.3 ± 8.0 dB SPL; *P* < 0.001) compared with wildtype mice (17.9 ± 6.7 dB SPL, 14/7 ears/animals). In contrast, click ABR thresholds were unaffected in all control groups (*Pax2::cre*: 13.8 ± 7.6 dB SPL, 16/8 ears/animals; Ca_v_1.3^+/*flex*^: 16.3 ± 4.3 dB SPL, 12/6 ears/animals; Ca_v_1.3^flex/-^: 20.4 ± 4.1 dB SPL; 14/7 ears/animals; *P* > 0.05; one-way ANOVA with Bonferroni *post hoc* test, Figure 8A). Frequency-dependent ABR (f-ABR) thresholds (Figure 8B) could only be measured in part of the cKO-Ca_v_1.3^flex/-^ mice analyzed (16/8 ears/animals, single data points) and were thus not included in the statistical analysis. f-ABR thresholds of *Pax2::cre* (15/8 ears/animals) and Ca_v_1.3^flex/-^ (14/7 ears/animals) significantly differed from wildtype mice (14/7 ears/animals; two-way ANOVA with Bonferroni *post hoc* test, effect of genotype: *P* < 0.001). Specifically, thresholds were increased in Ca_v_1.3^flex/-^ control mice (*P* < 0.001), reaching significance at all frequencies except at 22.6 kHz; and slightly reduced in *Pax2::cre* mice (*P* = 0.001), reaching significance at 2 and 8 kHz.

Averaged ABR waveforms of Ca_v_1.3^flex/-^ controls had smaller amplitudes than in wildtype mice for click stimuli 40 dB above threshold (Figure 8C). Growth functions of peak-to-peak amplitudes showed a significant reduction of all waves in Ca_v_1.3^flex/-^ control (14/7 ears/animals) compared with wildtype (wave I, IV: 14/7 ears/animals; wave II, III: 12/6 ears/animals, Figure 8D), revealed by a regression analysis of the smaller slopes of fits to the amplitudes as a function of level above threshold of wave I to IV (wave I, *P* < 0.001; wave II, *P* = 0.015; wave III, *P* < 0.001; wave IV, *P* = 0.027; *MWU* test) and a smaller *y*-axis intercept value of wave I (*MWU* test, *P* = 0.002). Growth functions of latencies, calculated as time between stimulus application and the negative peak of the respective wave, were not significantly altered for all waves (I – IV) in Ca_v_1.3^flex/-^ control mice compared with the wildtype (Figure 8E).

Finally, we tested the function of the cochlear amplifier including OHC electromotility by measuring DPOAEs (Figure 8F). Mean 2f1–f2 DPOAE maximum amplitudes averaged between 10 and 18 kHz in 0.5 kHz steps were strongly reduced in cKO-Ca_v_1.3^flex/-^ mice (12/7 ears/animals, *P* < 0.001, Kruskal–Wallis Test, effect of genotype) but unaffected in all control groups (wildtype: 14/7 ears/animals; *Pax2::cre*: 16/8 ears/animals; Ca_v_1.3^+/*flex*^: 12/6 ears/animals; Ca_v_1.3^flex/-^: 14/7 ears/animals).

In summary, cochlea-specific deletion of Ca_v_1.3 in cKO-Ca_v_1.3^flex/-^ mice resulted in highly elevated ABR thresholds and strongly reduced DPOAEs, reflecting profound hearing loss. Moreover, in Ca_v_1.3^flex/-^ control mice the reduction of mean IHC *I*_Ba_ amplitude to 42% and of mean Ca_v_1.3 cluster size to 73% is accompanied by increases in f-ABR thresholds up to 10 - 20 dB and strongly reduced amplitudes of ABR waves I to IV. Notably, click-ABR thresholds and DPOAEs were not affected in Ca_v_1.3^flex/-^ control mice.

## Discussion

Here we show that cochlea-specific ablation of Ca_v_1.3 channels resulted in an IHC phenotype replicating that of systemic Ca_v_1.3^-/-^ mice. For obtaining a cochlea-specific Ca_v_1.3 knockout mouse, we started with a conditional Ca_v_1.3^flex/flex^ mouse model that was crossed with a cochlea-specific Cre line, *Pax2::cre*. When determining Ca^2+^ channel currents through Ca_v_1.3 channels we found inefficient switch of both *flex* alleles in mice aged 3 weeks. In addition, IHCs of the basal cochlear turn from cKO-Ca_v_1.3^flex/flex^ mice were largely degenerated at 4–6 weeks of age, but not in cKO-Ca_v_1.3^flex/-^ mice. Moreover, it turned out that the *flex* allele itself had an impact on Ca_v_1.3 expression in IHCs. These obstacles demanded an in-depth analysis of the problems of the conditional mouse models used before drawing conclusions on the auditory phenotype caused by cochlea-specific deletion of Ca_v_1.3.

### Conditional Tissue-Specific Knockout Mice – Benefits and Pitfalls

Satheesh et al. (2012) were the first to analyze a conditional Ca_v_1.3 knockout mouse model with deletion in the auditory brainstem, the *Egr2::cre;Cacna1d-eGFP^flex/flex^* mouse. There, eGFP fluorescence was not detectable in unfixed brain tissue (Bartels, 2009), most likely due to the much lower abundance of eGFP/Ca_v_1.3 in these brainstem nuclei compared with IHCs. In the present study, the *flex* allele had the advantage that successful deletion of Ca_v_1.3 in IHCs resulted in eGFP fluorescence that could be judged semi-quantitatively at the cellular level. Moreover, the presence of individual IHCs where the *flex* allele was not switched enabled us to use these cells as positive controls, e.g., for Ca_v_1.3 immunolabeling within one specimen.

An unwanted side effect of transgenic animals is unexpected germline expression of Cre recombinase resulting in embryonal recombination of *loxP* sites that might even occur in Cre-negative offspring carrying a *flex* or *lox* allele (Song and Palmiter, 2018). This can be monitored (i) by adapting the genotyping protocol to recognize excised or switched *lox* or *flex* alleles and (ii) in the *flex* switch system as eGFP expression in cells of Cre-negative *flex* control mice with one or two *flex* alleles.

Without the GFP reporter function, we might not have detected the incomplete recombination of *flex* alleles in IHCs. Partial recombination of floxed alleles, resulting in a mixture of cells with recombination of both, one or even no allele, is a frequent problem in conditional knockout mice (Saam and Gordon, 1999; Schulz et al., 2007; Weis et al., 2010). Lack of knowledge about the amount of successful cellular deletion events may lead to wrong conclusions caused by residual functions contributed by non-knockout cells. In this study, about 10% of the IHCs in cKO-Ca_v_1.3^flex/-^ mice carried an unswitched *flex* allele resulting in residual hearing function compared with complete deafness of Ca_v_1.3^-/-^ mice (Platzer et al., 2000; Dou et al., 2004).

Our attempt to increase the success rate of Cre in switching the *flex* alleles by replacing one *flex* by a constitutive knockout (“-”) allele resulted in a higher ratio of true knockout IHCs in cKO-Ca_v_1.3^flex/-^ compared with cKO-Ca_v_1.3^flex/flex^ mice (cf. Figure 1, 2) at 3 weeks of age. However, this difference was no longer present 2 weeks later (Table 2), indicating that Cre managed to switch most *flex* alleles by this time point. An alternative approach to increase the recombination rate of *loxP* sites would be to increase expression of Cre recombinase (Schnütgen et al., 2003; Schulz et al., 2007) using *Pax2::cre/cre* instead of *Pax2::cre/*+ mice. However, high Cre expression levels on the other hand might increase the risk of possible side effects. High levels of Cre expression in α-myosin heavy chain-Cre mice have for example been demonstrated to be cardiotoxic causing altered cardiac function, DNA damage and inflammation (Bhandary and Robbins, 2015; Pugach et al., 2015). A dose dependence of Cre toxicity has been confirmed in cell culture titration experiments (Loonstra et al., 2001; Baba et al., 2005).

In summary, due to ambiguous eGFP expression in cKO-Ca_v_1.3^flex/flex^ mice when only one *flex* allele was switched resulting in heterozygous IHCs that still produced Ca_v_1.3, we decided to further use cKO-Ca_v_1.3^flex/-^ mice, where eGFP expression was a reliable marker of Ca_v_1.3 ablation. Since incomplete recombination of both *flex* (or *lox*) alleles is likely to be a general problem in conditional mice, a combination with a systemic knockout allele (*flex/–* or *lox/–*) should be used if possible.

### Toxicity of Excessive eGFP

Degeneration of IHCs in the basal cochlear turn as early as P25 in cKO-Ca_v_1.3^flex/flex^ but not Ca_v_1.3^-/-^ mice suggests toxicity of excessive eGFP. Furthermore, direct fluorescence of eGFP can be seen in non-fixed IHCs (this study) but not in the auditory brainstem (Bartels, 2009), further indicating particularly high expression of Ca_v_1.3 in wildtype and eGFP in cKO-Ca_v_1.3^flex/flex^ IHCs, respectively. GFP toxicity has been demonstrated in cell lines where its expression induced apoptosis (Liu et al., 1999) or inhibited polyubiquitination (Baens et al., 2006). In mice, neuronal expression of yellow fluorescent protein induced multiple dose-dependent stress responses (Comley et al., 2011). A possible cause for these damaging effects is that the pre-mature, colorless form of eGFP, which is present in variable proportions in GFP-expressing cells, produces the free radical O2^●-^ and hydrogen peroxide (H_2_O_2_) under consumption of NAD(P)H (Ganini et al., 2017). Such a GFP-induced oxidative stress may explain why only IHCs of cKO-Ca_v_1.3^flex/flex^ mice but not of cKO-Ca_v_1.3^flex/-^ degenerated because of the higher dose of eGFP produced by two *flex* alleles.

### Side Effects of Gene-Targeted Alleles Without Gene Deletion by Cre Recombinase

In conditional models, the modifications of the target gene should not affect its function unless recombined by Cre. In the study of Satheesh et al. (2012), who first described the Ca_v_1.3-*flex* model, Ca^2+^ currents were not analyzed. Normal ABR thresholds of Ca_v_1.3^flex/flex^ control mice led the authors to the conclusion that unswitched *flex* alleles did not affect Ca_v_1.3 channel function. Since Ca_v_1.3 channels mediate the majority of Ca^2+^ current in IHCs, the present study provided the unique opportunity to analyze potential side effects of the *flex* construct in detail by measuring Ca^2+^ channel currents and quantitatively analyzing Ca_v_1.3 protein clusters. We found reduced *I*_Ba_ amplitudes and a lower amount of Ca_v_1.3 protein in IHCs of cre-negative Ca_v_1.3^flex/flex^ and Ca_v_1.3^flex/-^ control mice demonstrating that the unswitched *flex* allele did not fully replace the wildtype function. As has been shown before, a considerable reduction or increase in peak Ca^2+^ current amplitudes of IHCs has only minor effects on click ABR thresholds (Scharinger et al., 2015; Fell et al., 2016), which can be misleading when used as the only method to assess the function of IHCs. Here, frequency-dependent ABR thresholds were increased by 10–20 dB at most frequencies in Ca_v_1.3^flex/-^ control mice upon reduction of *I_Ba_* to 42% of the wildtype value, which is in accordance to threshold increases of 5 – 20 dB in null mutants of the auxiliary α_2_δ2 Ca^2+^ channel subunit causing reductions of *I_Ba_* to 60–70% (Fell et al., 2016). The most prominent consequence of *I_Ba_* reduction with respect to hearing function of Ca_v_1.3^flex/-^ control mice are the reduced growth functions of peak-to-peak amplitudes of the ABR waves, especially wave I, indicating strongly reduced IHC output at all levels above threshold. It should be kept in mind that both click and frequency-specific ABR thresholds are determined by only one afferent fiber type, the low threshold, high spontaneous rate fibers, whereas growth functions of ABR amplitudes cover the activity of all (high, medium and low spontaneous rate) afferent fiber types (Kiang et al., 1965; Liberman, 1978, 1982; Petitpré et al., 2018; Shrestha et al., 2018; Sun et al., 2018).

The question arises as to why *I_Ba_* was reduced in IHCs of Cre-negative mice containing at least one *flex* allele (Figure 6). For generating the conditional Ca_v_1.3 mouse, the *Cacna1d-eGFP^flex^* construct was placed outside and a few hundred base pairs up- and downstream of the coding regions of exon 2 in the *Cacna1d* gene to avoid unintended manipulation of regulatory elements flanking the exon (Bartels, 2009). This insertion might have disrupted unknown regulatory elements and thus reduced the expression level of the channel. Moreover, *I_Ba_* gating properties were altered in Ca_v_1.3^flex/-^ control mice, suggesting that insertion of the *flex* construct might have affected splicing of *Cacna1d* mRNA (Bock et al., 2011; Scharinger et al., 2015).

### The IHC and Auditory Phenotype Following Systemic Versus Cochlea-Specific Deletion of Ca_v_1.3 Channels

The ablation of Ca_v_1.3 channels before birth in cKO-Ca_v_1.3^flex/-^ mice caused an IHC phenotype similar to that of Ca_v_1.3^-/-^ mice, including persistent expression of SK2, lack of BK expression in apical-turn IHCs, and a reduced cell size (Brandt et al., 2003; Glueckert et al., 2003; Nemzou et al., 2006).

Until the onset of hearing, SK2 channels mediate efferent inhibition of IHCs via α9α10 Ca^2+^-permeable nicotinic acetylcholine receptors (nAChRs) (Oliver et al., 2000; Elgoyhen et al., 2001), The origin of these efferent fibers lies in cholinergic neurons in the SOC. Shortly after birth, neurons of the auditory brainstem are spontaneously active and undergo a developmental program including synaptic pruning and establishment of tonotopy (Blankenship and Feller, 2010; Clause et al., 2014). The spiking pattern of SOC neurons is modulated by ascending information from the cochlea, where IHCs produce spontaneous Ca^2+^ action potentials, which are synchronized by Ca^2+^ waves in the transient Kölliker’s organ (Tritsch and Bergles, 2010; Johnson et al., 2011, 2017; Sendin et al., 2014; Eckrich et al., 2018; Mammano and Bortolozzi, 2018). In turn, efferent inhibition from the SOC closes a feedback loop by shaping the spontaneous activity of IHCs (Guinan, 2006; Frank and Goodrich, 2018). In the systemic Ca_v_1.3 knockout mouse both SOC neurons and IHCs lack Ca_v_1.3 currents (Platzer et al., 2000; Hirtz et al., 2011), the latter of which as a consequence cannot produce action potentials (Brandt et al., 2003). In the SOC of Ca_v_1.3^-/-^ mice, depolarization-induced spiking of lateral superior olive (LSO) neurons was changed from a single to a multiple firing pattern due to a reduction in K_v_1.2 channels (Hirtz et al., 2011). This was most likely caused by the specific lack of Ca_v_1.3 channels in brainstem neurons despite intact cochlear expression as confirmed in brainstem-specific Ca_v_1.3 knockout mice (Satheesh et al., 2012). In the present study with SOC neurons expressing Ca_v_1.3 channels, the phenotype of Ca_v_1.3-deficient IHCs from cKO-Ca_v_1.3^flex/-^ mice (lack of BK channels, persistence of SK2 channels, smaller cell size) was very similar to that of systemic Ca_v_1.3^-/-^ mice. Therefore a potentially altered feedback signaling by Ca_v_1.3-deficient SOC neurons onto IHCs cannot be causative for the IHC phenotype of Ca_v_1.3^-/-^ mice. Nevertheless, the spiking pattern of SOC neurons and, thus, efferent signaling back to immature IHCs might still be altered due to the loss of afferent activation by IHCs. In α9- and α10-nAChR knockout mice, maturation of IHC K^+^ channels was normal despite the complete lack of cholinergic efferent input from SOC neurons (Gomez-Casati et al., 2009; Johnson et al., 2013b). In summary, maturation of the IHC’s K^+^ channel composition is mainly controlled by intrinsic Ca^2+^ signaling within the IHC and does not depend on Ca_v_1.3 expression in the SOC exerting efferent feedback.

### BK Channel Expression in Basal-Turn IHCs of Ca_v_1.3^-/-^ and Cre-Negative IHCs of cKO-Ca_v_1.3^flex/-^ Mice

We found residual BK labeling in IHCs of the basal but not the apical cochlear turn of Ca_v_1.3^-/-^ mice. So far it is unknown why BK protein is missing in IHCs of Ca_v_1.3^-/-^ mice along most of the cochlear length (Brandt et al., 2003) despite expression of the respective *Kcnma1* mRNA (Nemzou et al., 2006).

The faint and dot-like BK labeling in GFP-negative IHCs of cKO-Ca_v_1.3^flex/-^ mice clearly differed from the large BK patches found in IHCs of wildtype and control Ca_v_1.3^flex/-^ mice (Figure 5F). Assuming that Cre is not active in these IHCs their phenotype should be the same as that of Ca_v_1.3^flex/-^ controls (Figure 5D) but this was not the case. We can thus exclude that the reduced BK expression was caused by the incomplete wildtype function of the unswitched *flex* allele. But what are the differences between IHCs of Ca_v_1.3^flex/-^ controls and GFP-negative IHCs of cKO-Ca_v_1.3^flex/-^ mice? Differences intrinsic to the IHCs are: (i) Presence of the *Pax2::cre* allele at an unknown location in the genome, which might interfere with modulatory sequences affecting BK expression; (ii) Cre might be expressed in these IHCs without switching the *flex* allele, but it could still interfere with BK expression. Alternatively, a factor extrinsic to the IHC might be causing the reduced BK expression. GFP-negative IHCs of cKO-Ca_v_1.3^flex/-^ mice are surrounded by Ca_v_1.3-deficient, electrically silent IHCs, whereas neonatal IHCs produce Ca^2+^ action potentials in wildtype and presumably Ca_v_1.3^flex/-^ mice (Brandt et al., 2003). This activity causes periodic efflux of K^+^ ions from the IHCs, which depolarizes neighboring phalangeal cells and IHCs, thereby amplifying and synchronizing Ca^2+^ AP activity (Wang et al., 2015; Eckrich et al., 2018). In summary, impaired expression of BK channels in solitary GFP-negative IHCs surrounded by true Ca_v_1.3 knockout IHCs of cKO-Ca_v_1.3^flex/-^ mice may result from a lack of mutual activation and synchronization of Ca^2+^ AP activity among IHCs during the critical developmental period. It would be interesting to analyze whether the Ca^2+^ action potential activity in GFP-negative IHCs of cKO-Ca_v_1.3^flex/-^ is altered compared to Ca_v_1.3^flex/-^ controls.

## Data Availability

The datasets generated for this study are available on request to the corresponding author.

## Ethics Statement

All experiments were carried out in accordance with the European Communities Council Directive (86/609/EEC) and approved by the regional board for scientific animal experiments of the Saarland, Germany. Additional ethics approval was not required according to the local and national guidelines.

## Author Contributions

SE and JE conceived and designed the study. SE, DH, KS, KB, KF, and SM acquired the data. SE, JE, DH, KB, SM, GW, and BS drafted the article. JE contributed to funding acquisition and project administration. All authors analyzed and interpreted the data.

## Conflict of Interest Statement

The authors declare that the research was conducted in the absence of any commercial or financial relationships that could be construed as a potential conflict of interest.

## References

[B1] Baba Y. Nakano M. Yamada Y. Saito I. Kanegae Y. (2005). Practical range of effective dose for cre recombinase-expressing recombinant adenovirus without cell toxicity in mammalian cells. *Microbiol. Immunol.* 49 559–570. 10.1111/j.1348-0421.2005.tb03753.x 15965304

[B2] Babola T. A. Li S. Gribizis A. Lee B. J. Issa J. B. Wang H. C. (2018). Homeostatic control of spontaneous activity in the developing auditory system. *Neuron* 99 511.e5–524.e5. 10.1016/j.neuron.2018.07.004 30077356 PMC6100752

[B3] Baens M. Noels H. Broeckx V. Hagens S. Fevery S. Billiau A. D. (2006). The dark side of EGFP: defective polyubiquitination. *PLoS One* 1:e54. 10.1371/journal.pone.0000054 17183684 PMC1762387

[B4] Baig S. M. Koschak A. Lieb A. Gebhart M. Dafinger C. Nürnberg G. (2011). Loss of Cav1.3 (CACNA1D) function in a human channelopathy with bradycardia and congenital deafness. *Nat. Neurosci.* 14 77–84. 10.1038/nn.2694 21131953

[B5] Bartels K. (2009). *Conditional Knockout of the L-Type Voltage-Gated Calcium Channel CaV1.3 via the FLEX Switch.* Thesis Heidelberg: Ruprecht-Karls-Universität Heidelberg, 10.11588/heidok.00010021

[B6] Bhandary B. Robbins J. (2015). Giving credence to controls: avoiding the false phenotype. *J. Mol. Cell. Cardiol.* 86 136–137. 10.1016/j.yjmcc.2015.07.007 26235056 PMC4804197

[B7] Blankenship A. G. Feller M. B. (2010). Mechanisms underlying spontaneous patterned activity in developing neural circuits. *Nat. Rev. Neurosci.* 11 18–29. 10.1038/nrn2759 19953103 PMC2902252

[B8] Bock G. Gebhart M. Scharinger A. Jangsangthong W. Busquet P. Poggiani C. (2011). Functional properties of a newly identified c-terminal splice variant of Cav1.3 L-type Ca2+ channels. *J. Biol. Chem.* 286 42736–42748. 10.1074/jbc.M111.269951 21998310 PMC3234942

[B9] Brandt A. Striessnig J. Moser T. (2003). CaV1.3 channels are essential for development and presynaptic activity of cochlear inner hair cells. *J. Neurosci.* 23 10832–10840. 10.1523/JNEUROSCI.23-34-10832.2003 14645476 PMC6740966

[B10] Burton Q. Cole L. K. Mulheisen M. Chang W. Wu D. K. (2004). The role of Pax2 in mouse inner ear development. *Dev. Biol.* 272 161–175. 10.1016/j.ydbio.2004.04.024 15242798

[B11] Clause A. Kim G. Sonntag M. Weisz C. J. C. Vetter D. E. Rübsamen R. (2014). The precise temporal pattern of prehearing spontaneous activity is necessary for tonotopic map refinement. *Neuron* 82 822–835. 10.1016/j.neuron.2014.04.001 24853941 PMC4052368

[B12] Comley L. H. Wishart T. M. Baxter B. Murray L. M. Nimmo A. Thomson D. (2011). Induction of cell stress in neurons from transgenic mice expressing yellow fluorescent protein: implications for neurodegeneration research. *PLoS One* 6:e17639. 10.1371/journal.pone.0017639 21408118 PMC3050905

[B13] Dou H. Vazquez A. E. Namkung Y. Chu H. Cardell E. L. Nie L. (2004). Null mutation of alpha1D Ca2+ channel gene results in deafness but no vestibular defect in mice. *J. Assoc. Res. Otolaryngol.* 5 215–226. 10.1007/s10162-003-4020-3 15357422 PMC2538408

[B14] Eckrich T. Blum K. Milenkovic I. Engel J. (2018). Fast Ca2+ transients of inner hair cells arise coupled and uncoupled to Ca2+ waves of inner supporting cells in the developing mouse cochlea. *Front. Mol. Neurosci.* 11:264. 10.3389/fnmol.2018.00264 30104958 PMC6077211

[B15] Elgoyhen A. B. Vetter D. E. Katz E. Rothlin C. V. Heinemann S. F. Boulter J. (2001). α10: a determinant of nicotinic cholinergic receptor function in mammalian vestibular and cochlear mechanosensory hair cells. *Proc. Natl. Acad. Sci. U.S.A.* 98 3501–3506. 10.1073/pnas.051622798 11248107 PMC30682

[B16] Engel J. Braig C. Ruttiger L. Kuhn S. Zimmermann U. Blin N. (2006). Two classes of outer hair cells along the tonotopic axis of the cochlea. *Neuroscience* 143 837–849. 10.1016/j.neuroscience.2006.08.060 17074442

[B17] Fell B. Eckrich S. Blum K. Eckrich T. Hecker D. Obermair G. J. (2016). α2δ2 controls the function and trans-synaptic coupling of Cav1.3 channels in mouse inner hair cells and is essential for normal hearing. *J. Neurosci.* 36 11024–11036. 10.1523/JNEUROSCI.3468-14.2016 27798183 PMC6705655

[B18] Frank M. M. Goodrich L. V. (2018). Talking back: development of the olivocochlear efferent system. *Wiley Interdiscip. Rev. Dev. Biol.* 7:e324. 10.1002/wdev.324 29944783 PMC6185769

[B19] Ganini D. Leinisch F. Kumar A. Jiang J. Tokar E. J. Malone C. C. (2017). Fluorescent proteins such as eGFP lead to catalytic oxidative stress in cells. *Redox Biol.* 12 462–468. 10.1016/j.redox.2017.03.002 28334681 PMC5362137

[B20] Glowatzki E. Fuchs P. A. (2000). Cholinergic synaptic inhibition of inner hair cells in the neonatal mammalian cochlea. *Science* 288 2366–2368. 10.1126/science.288.5475.2366 10875922

[B21] Glueckert R. Wietzorrek G. Kammen-Jolly K. Scholtz A. Stephan K. Striessnig J. (2003). Role of class D L-type Ca2+ channels for cochlear morphology. *Hear. Res.* 178 95–105. 10.1016/S0378-5955(03)00054-6 12684182

[B22] Gomez-Casati M. E. Wedemeyer C. Taranda J. Lipovsek M. Dalamon V. Elgoyhen A. B. (2009). Electrical properties and functional expression of ionic channels in cochlear inner hair cells of mice lacking the alpha10 nicotinic cholinergic receptor subunit. *J. Assoc. Res. Otolaryngol.* 10 221–232. 10.1007/s10162-009-0164-0 19252947 PMC2674205

[B23] Guinan J. J. (2006). Olivocochlear efferents: anatomy, physiology, function, and the measurement of efferent effects in humans. *Ear Hear.* 27 589–607. 10.1097/01.aud.0000240507.83072.e7 17086072

[B24] Hirtz J. J. Boesen M. Braun N. Deitmer J. W. Kramer F. Lohr C. (2011). Cav1.3 calcium channels are required for normal development of the auditory brainstem. *J. Neurosci.* 31 8280–8294. 10.1523/JNEUROSCI.5098-10.2011 21632949 PMC6622878

[B25] Hirtz J. J. Braun N. Griesemer D. Hannes C. Janz K. Löhrke S. (2012). Synaptic refinement of an inhibitory topographic map in the auditory brainstem requires functional CaV1.3 calcium channels. *J. Neurosci.* 32 14602–14616. 10.1523/JNEUROSCI.0765-12.2012 23077046 PMC6621425

[B26] Jae Huh W. Mysorekar I. U. Mills J. C. (2010). Inducible activation of Cre recombinase in adult mice causes gastric epithelial atrophy, metaplasia, and regenerative changes in the absence of “floxed” alleles. *Am. J. Physiol. Gastrointest. Liver Physiol.* 299 G368–G380. 10.1152/ajpgi.00021.2010 20413717 PMC3774481

[B27] Janbandhu V. Moik D. Fässler R. (2014). Cre recombinase induces DNA damage and tetraploidy in the absence of LoxP sites. *Cell Cycle* 13 462–470. 10.4161/cc.27271 24280829 PMC3956542

[B28] Johnson S. L. Ceriani F. Houston O. Polishchuk R. Polishchuk E. Crispino G. (2017). Connexin-mediated signaling in nonsensory cells is crucial for the development of sensory inner hair cells in the mouse cochlea. *J. Neurosci.* 37 258–268. 10.1523/JNEUROSCI.2251-16.2016 28077706 PMC5242392

[B29] Johnson S. L. Eckrich T. Kuhn S. Zampini V. Franz C. Ranatunga K. M. (2011). Position-dependent patterning of spontaneous action potentials in immature cochlear inner hair cells. *Nat. Neurosci.* 14 711–717. 10.1038/nn.2803 21572434 PMC3103712

[B30] Johnson S. L. Kuhn S. Franz C. Ingham N. Furness D. N. Knipper M. (2013a). Presynaptic maturation in auditory hair cells requires a critical period of sensory-independent spiking activity. *Proc. Natl. Acad. Sci. U.S.A.* 110 8720–8725. 10.1073/pnas.1219578110 23650376 PMC3666720

[B31] Johnson S. L. Wedemeyer C. Vetter D. E. Adachi R. Holley M. C. Elgoyhen A. B. (2013b). Cholinergic efferent synaptic transmission regulates the maturation of auditory hair cell ribbon synapses. *Open Biol.* 3:130163. 10.1098/rsob.130163 24350389 PMC3843824

[B32] Kiang N. Y. Pfeiffer R. R. Warr W. B. Backus A. S. (1965). Stimulus coding in the cochlear nucleus. *Trans. Am. Otol. Soc.* 53 35–58. 5834666

[B33] Kros C. J. Ruppersberg J. P. Rüsch A. (1998). Expression of a potassium current in inner hair cells during development of hearing in mice. *Nature* 394 281–284. 10.1038/28401 9685158

[B34] Lawoko-Kerali G. Rivolta M. N. Holley M. C. (2001). Expression of the transcription factors GATA3 and Pax2 during development of the mammalian inner ear. *J. Comp. Neurol.* 442 378–391. 10.1002/cne.10088 11793341

[B35] Liberman M. C. (1978). Auditory-nerve response from cats raised in a low-noise chamber. *J. Acoust. Soc. Am.* 63 442–455. 10.1121/1.381736 670542

[B36] Liberman M. C. (1982). Single-neuron labeling in the cat auditory nerve. *Science* 216 1239–1241. 10.1126/science.7079757 7079757

[B37] Liu H.-S. Jan M.-S. Chou C.-K. Chen P.-H. Ke N.-J. (1999). Is green fluorescent protein toxic to the living cells? *Biochem. Biophys. Res. Commun.* 260 712–717. 10.1006/bbrc.1999.0954 10403831

[B38] Loonstra A. Vooijs M. Beverloo H. B. Allak B. A. Drunen E. van Kanaar R. (2001). Growth inhibition and DNA damage induced by Cre recombinase in mammalian cells. *Proc. Natl. Acad. Sci.* 98 9209–9214. 10.1073/pnas.161269798 11481484 PMC55399

[B39] Mammano F. Bortolozzi M. (2018). Ca2+ signaling, apoptosis and autophagy in the developing cochlea: milestones to hearing acquisition. *Cell Calcium* 70 117–126. 10.1016/j.ceca.2017.05.006 28578918

[B40] Marcotti W. Johnson S. L. Kros C. J. (2004). A transiently expressed SK current sustains and modulates action potential activity in immature mouse inner hair cells. *J. Physiol.* 560 691–708. 10.1113/jphysiol.2004.072868 15331671 PMC1665291

[B41] Marcotti W. Johnson S. L. Rusch A. Kros C. J. (2003). Sodium and calcium currents shape action potentials in immature mouse inner hair cells. *J. Physiol.* 552 743–761. 10.1113/jphysiol.2003.043612 12937295 PMC2343463

[B42] Nemzou N. R. M. Bulankina A. V. Khimich D. Giese A. Moser T. (2006). Synaptic organization in cochlear inner hair cells deficient for the CaV1.3 (α1D) subunit of L-type Ca2+ channels. *Neuroscience* 141 1849–1860. 10.1016/j.neuroscience.2006.05.057 16828974

[B43] Ohyama T. Groves A. K. (2004). Generation of Pax2-Cre mice by modification of a Pax2 bacterial artificial chromosome. *Genesis* 38 195–199. 10.1002/gene.20017 15083520

[B44] Oliver D. Klocker N. Schuck J. Baukrowitz T. Ruppersberg J. P. Fakler B. (2000). Gating of Ca2+-activated K+ channels controls fast inhibitory synaptic transmission at auditory outer hair cells. *Neuron* 26 595–601. 10.1016/S0896-6273(00)81197-6 10896156

[B45] Oliver D. Knipper M. Derst C. Fakler B. (2003). Resting potential and submembrane calcium concentration of inner hair cells in the isolated mouse cochlea are set by KCNQ-type potassium channels. *J. Neurosci.* 23 2141–2149. 10.1523/JNEUROSCI.23-06-02141.2003 12657673 PMC6742048

[B46] Petitpré C. Wu H. Sharma A. Tokarska A. Fontanet P. Wang Y. (2018). Neuronal heterogeneity and stereotyped connectivity in the auditory afferent system. *Nat. Commun.* 9:3691. 10.1038/s41467-018-06033-3 30209249 PMC6135759

[B47] Platzer J. Engel J. Schrott-Fischer A. Stephan K. Bova S. Chen H. (2000). Congenital deafness and sinoatrial node dysfunction in mice lacking class D L-type Ca2+ channels. *Cell* 102 89–97. 10.1016/s0092-8674(00)00013-1 10929716

[B48] Pugach E. K. Richmond P. A. Azofeifa J. G. Dowell R. D. Leinwand L. A. (2015). Prolonged Cre expression driven by the α-myosin heavy chain promoter can be cardiotoxic. *J. Mol. Cell. Cardiol.* 86 54–61. 10.1016/j.yjmcc.2015.06.019 26141530 PMC4558343

[B49] Saam J. R. Gordon J. I. (1999). Inducible gene knockouts in the small intestinal and colonic epithelium. *J. Biol. Chem.* 274 38071–38082. 10.1074/jbc.274.53.38071 10608876

[B50] Sachs L. (1999). *Angewandte Statistik. Anwendung statistischer Methoden.* Berlin: Springer.

[B51] Satheesh S. V. Kunert K. Rüttiger L. Zuccotti A. Schönig K. Friauf E. (2012). Retrocochlear function of the peripheral deafness gene Cacna1d. *Hum. Mol. Genet.* 21 3896–3909. 10.1093/hmg/dds217 22678062

[B52] Scharinger A. Eckrich S. Vandael D. H. Schönig K. Koschak A. Hecker D. (2015). Cell-type-specific tuning of Cav1.3 Ca2+-channels by a C-terminal automodulatory domain. *Front. Cell. Neurosci.* 9:309 10.3389/fncel.2015.00309 PMC4547004 26379493

[B53] Schindelin J. Arganda-Carreras I. Frise E. Kaynig V. Longair M. Pietzsch T. (2012). Fiji: an open-source platform for biological-image analysis. *Nat. Methods* 9 676–682. 10.1038/nmeth.2019 22743772 PMC3855844

[B54] Schnütgen F. Doerflinger N. Calléja C. Wendling O. Chambon P. Ghyselinck N. B. (2003). A directional strategy for monitoring Cre-mediated recombination at the cellular level in the mouse. *Nat. Biotechnol.* 21 562–565. 10.1038/nbt811 12665802

[B55] Schulz T. J. Glaubitz M. Kuhlow D. Thierbach R. Birringer M. Steinberg P. (2007). Variable expression of cre recombinase transgenes precludes reliable prediction of tissue-specific gene disruption by tail-biopsy genotyping. *PLoS One* 2:e1013. 10.1371/journal.pone.0001013 17925861 PMC1995755

[B56] Sendin G. Bourien J. Rassendren F. Puel J.-L. Nouvian R. (2014). Spatiotemporal pattern of action potential firing in developing inner hair cells of the mouse cochlea. *Proc. Natl. Acad. Sci. U.S.A.* 111 1999–2004. 10.1073/pnas.1319615111 24429348 PMC3918831

[B57] Shrestha B. R. Chia C. Wu L. Kujawa S. G. Liberman M. C. Goodrich L. V. (2018). Sensory neuron diversity in the inner ear is shaped by activity. *Cell* 174 1229.e17–1246.e17. 10.1016/j.cell.2018.07.007 30078709 PMC6150604

[B58] Simmons D. D. (2002). Development of the inner ear efferent system across vertebrate species. *J. Neurobiol.* 53 228–250. 10.1002/neu.10130 12382278

[B59] Song A. J. Palmiter R. D. (2018). Detecting and avoiding problems when using the Cre–lox system. *Trends Genet.* 34 333–340. 10.1016/j.tig.2017.12.008 29336844 PMC5910175

[B60] Sun S. Babola T. Pregernig G. So K. S. Nguyen M. Su S.-S. M. (2018). Hair cell mechanotransduction regulates spontaneous activity and spiral ganglion subtype specification in the auditory system. *Cell* 174 1247.e–1263.e. 10.1016/j.cell.2018.07.008 30078710 PMC6429032

[B61] Tritsch N. X. Bergles D. E. (2010). Developmental regulation of spontaneous activity in the Mammalian cochlea. *J. Neurosci.* 30 1539–1550. 10.1523/JNEUROSCI.3875-09.2010 20107081 PMC2814371

[B62] Wang H. C. Lin C.-C. Cheung R. Zhang-Hooks Y. Agarwal A. Ellis-Davies G. (2015). Spontaneous activity of cochlear hair cells triggered by fluid secretion mechanism in adjacent support cells. *Cell* 163 1348–1359. 10.1016/j.cell.2015.10.070 26627734 PMC4671825

[B63] Weis B. Schmidt J. Lyko F. Linhart H. G. (2010). Analysis of conditional gene deletion using probe based real-time PCR. *BMC Biotechnol.* 10:75. 10.1186/1472-6750-10-75 20950424 PMC2966447

[B64] Willaredt M. A. Ebbers L. Nothwang H. G. (2014). Central auditory function of deafness genes. *Hear. Res.* 312 9–20. 10.1016/j.heares.2014.02.004 24566090

[B65] Zuccotti A. Kuhn S. Johnson S. L. Franz C. Singer W. Hecker D. (2012). Lack of brain-derived neurotrophic factor hampers inner hair cell synapse physiology, but protects against noise-induced hearing loss. *J. Neurosci.* 32 8545–8553. 10.1523/JNEUROSCI.1247-12.2012 22723694 PMC6620992

